# A novel *ex vivo* porcine model of acid-induced esophageal damage for preliminary functional evaluations of anti-gastroesophageal reflux disease medical devices

**DOI:** 10.14202/vetworld.2020.2728-2735

**Published:** 2020-12-21

**Authors:** Domenico Ventrella, Roberta Salaroli, Alberto Elmi, Giacomo Carnevali, Monica Forni, Fabio Baldi, Maria Laura Bacci

**Affiliations:** 1 Department of Veterinary Medical Sciences, University of Bologna, Ozzano dell’Emilia (BO), Italy; 2 Center for the Study of Diseases of the Esophagus, University of Bologna, Bologna, Italy; 3 GVM Care and Research, Cotignola (RA), Italy

**Keywords:** esophagus *ex vivo* model, Evans blue permeability assay, gastroesophageal reflux disease, multichannel intraluminal impedance, pig

## Abstract

**Aim:**

The aim of the study was to set up a porcine *ex vivo* model of acid-induced damage and to evaluate its performance by means of multichannel intraluminal impedance and pH (MII-pH) live recording, histology, and Evans blue (EB) permeability assay.

**Materials and Methods:**

Thirteen esophagi, collected at a slaughterhouse, were ablated of their sphincters, pinned upright on a support, and placed in a thermostatic hood at 37°C with two infusion tubes and an MII-pH probe inserted in the top end. Three esophagi (histology controls) were only left in the hood for 3.5 h before sampling, while the remaining organs underwent the experimental protocol including saline infusion and recovery recording, and acid solution infusion and recovery recording.

**Results:**

MII-pH analysis highlighted a significantly stronger decrease during acid infusion when compared to saline, but a better post-infusion recovery for saline solution. At the end of the protocol, MII was still statistically lower than baseline. The acid-damaged esophagi significantly absorbed more EB dye, and histology revealed strong mucosal exfoliation.

**Conclusion:**

The proposed model of esophageal acid damage seems to be repeatable, reliable, and achievable using organs collected at the slaughterhouse. MII recording proved to have good sensitivity in detecting mucosal alterations also in *ex vivo* trials.

## Introduction

Gastroesophageal reflux is a physiological event occurring during and after meals that becomes pathological, under the name of gastroesophageal reflux disease (GERD), when the reflux of stomach contents causes symptoms and complications [1]. Common GERD-associated symptoms are heartburn and regurgitation [2] while, in worst cases, other “extra-esophageal symptoms” such as chronic cough, aspiration pneumonia, pulmonary fibrosis, hoarseness, and dental erosions are reported [3]. The disease shows an estimated prevalence of 20-25% in industrialized countries, with an increasing rate mainly due to the diffusion of obesity [4], and has high economic impact, with both direct and indirect costs up to 10 billion dollars per year only in the United States [2,5]. Most patients affected by GERD experience resolution of symptoms on administration of proton-pump inhibitors (PPIs) [6], but these drugs do not seem to alleviate symptoms nor protect the esophageal mucosa in case of erosive lesions [2]. Based on the findings of conventional endoscopy and histopathological examination, GERD is categorized into three progressive stages: non-erosive reflux disease, reflux esophagitis, and Barrett’s esophagus [7,8]. As mucosal integrity is a pivotal factor in preventing inflammatory and erosive forms [9], new therapeutic approaches are focusing on drugs capable of being retained within the esophagus, thus coating and protecting its mucosal layer without inducing esophageal blockage [10]. Nowadays, a variety of formulas based on either hyaluronic acid and chondroitin sulfate [11] or sodium alginate [12] is available. When it comes to diagnosis of this pathology, different methods, such as PPI trials and endoscopic evaluation, can be used [13]. Nonetheless, it was recently proved how the most reliable diagnostic test is 24 h pH monitoring [14], as it allows for the quantification of acid exposure and correlation between symptoms and reflux episodes [2]. Moreover, as the type of the reflux can differ (acid, non-acid, liquid, or gas) depending on the variability of the gastric contents composition [15], pH monitoring can be combined with multichannel intraluminal impedance and pH (MII-pH) measurement to gain more info regarding the nature of the reflux itself [16]. Indeed, MII was first introduced to investigate the characteristics of the reflux in the esophagus [17] and can be used both as a diagnostic tool [15] and therapy outcome monitoring [9], and has also been proven to be indicative of mucosal lesions [18].

Many *ex vivo* models have been validated and optimized to investigate the esophageal coating potential of different medical devices [19-21], always using mucosal portions of porcine esophagi. Overall, porcine esophagi represent a good model to perform screening tests, as they can be easily collected at slaughterhouses and present more histological and morphological similarities with the human esophagus if compared to rodents [22]. While *ex vivo* models have been used to test the efficacy of medical devices through histological and pH analyses [11], the measurement of impedance in an *ex vivo* model is still lacking and may provide important new insight into experimental trials.

Therefore, the aim of this study was to set up an *ex vivo* porcine model of acid-induced damage and to evaluate its translatability and reproducibility by means of MII, pH, histology, and Evans blue (EB) permeability assay.

## Materials and Methods

### Ethical approval

The present work included tissues collected at the slaughterhouse from carcasses destined for human consumption, thus replacing killing animals for tissue sampling in accordance with the 3Rs; no animal was sacrificed solely for the purpose of this experiments therefore, no ethical approval was needed.

### Study period and location

The experiments were carried out from July 2017 to April 2018 at the Physiology Laboratories of the Department of Veterinary Medical Sciences of the University of Bologna (Ozzano dell’Emilia, Italy).

### Organs collection and preparation

**Figure-1:**
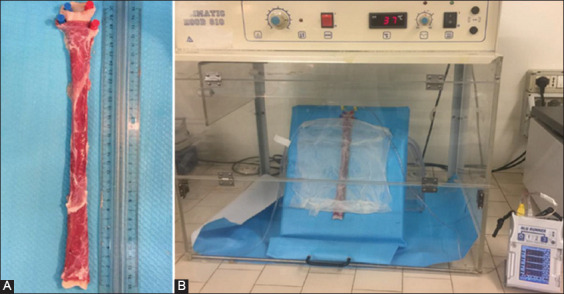
(A) Esophagus ablated of the upper and lower sphincters, cut to a final length of 30 cm and pinned to the support. (B) Experimental set up in the thermostatic hood.

Thirteen (n=13) swine esophagi were collected at a local slaughterhouse from commercially available European breed pigs and transferred within 2 h in cooled saline to the physiology labs of the Department of Veterinary Medical Sciences of the University of Bologna. On arrival, each esophagus was thoroughly rinsed with tap water (both inside and outside) and ablated of the cranial and caudal sphincters, maintaining a total length of 30 cm (Figure-1A). Only organs without visible lesions were used for experimental purposes. Organs were then pinned upright (cranial end at the top and caudal end at the bottom) on polystyrene support and placed, with an inclination of approximately 45°, in a thermostatic hood (Climatic Hood 810; ASAL s.r.l, Cernusco sul Naviglio, Italy) set at 37°C (Figure-1B), as previously described [11]. Organs were externally kept moist by means of wet tissue paper. Once positioned, two infusion tubes connected to dedicated pumps were inserted in the top end of the organ for the infusion of the different solutions. Finally, anion-sensitive field-effect transistor (ISFET) MII-pH probe (pHTip 1pH 8E, UNISENSOR AG, Attikon, Switzerland) was inserted in each esophagus for a total length of 25 cm; adherence to the mucosa was insured by applying a delicate pressure on the organs. Three esophagi, used as controls for histology, were only set up and left in the thermostatic hood for 3.5 h (overall time of the experimental protocol), before sampling.

### Solutions

To perform basal “wet” recordings, similar to water deglutition, commercially available sterile saline was used (NaCl 0.9 %; S.A.L.F. Spa Laboratorio Farmaceutico, Cenate Sotto, Italy), while to mimic acid gastroesophageal reflux, a 0.1 M hydrochloric acid solution was freshly prepared on the day of each trial (hydrochloric acid 37 % RPE, CARLO ERBA Reagents s.r.l, Cornaredo, Italy). Artificial saliva, used to simulate the physiological environment of the esophagus, was prepared as previously described [10]. All the solutions were used at room temperature.

### Experimental protocol

The experimental protocol, applied to 10 esophagi, is reported in Table-1. For the first 15 min, no infusion was performed to analyze baseline mucosal impedance and pH. Then, saline washing was started with an infusion rate of 600 mL/h and lasted for 15 min followed by 30 min of “dry” registration. Afterward, to induce the acid damage, acid solution (HCl 0.1 M) infusion was performed for 30 min at the same infusion rate followed by a final registration of 120 min. During the final period of registration, two saliva washings (10 mL infused in 1 min, each) were done at 45 and 90 min after the end of the acid treatment. For each infusion (saline, acid, and saliva), fluids were collected in a Petri dish placed under the organs and measured. The total duration of the experimental protocol was 3.5 h.

**Table-1:** Experimental protocol.

Time (min)	Procedures
0-15	Baseline recording
15-30	Saline infusion 600 mL/h
30-60	Post-saline recording
60-90	Acid solution infusion 600 mL/h
90-210	Post-acid recording
135	1^st^ saliva bolus
180	2^nd^ saliva bolus

### MII-pH

MII-pH was recorded for the entire duration of the protocol using a modified version of a commercial impedance recorder (BLU RUNNER; Menfis Biomedical s.r.l., Bologna, Italy). The disposable ISFET probes, with 1 distal pH sensor and 6 recording impedance channels, were calibrated (1 and 7 pH standard buffers, BIOTECH, Vesoul, France) and activated with tap water according to the manufacturer’s instruction before each use. At the end of each experiment, data regarding pH and impedance were exported using the software IMP/HS Dyno 3000. Five different time points were used for the inferential statistical analysis as shown in Table-2: The end of the baseline recording (t0), the end of the saline solution infusion (t1), the end of the post-saline infusion recording (t2), the end of the acid infusion (t3), and, finally, 30 min of post-acid infusion recording (t4).

**Table-2:** Time points for MII-pH analyses.

Time point	Event	Minute of recording
t0	End of baseline recording	15 min
t1	End of saline infusion	30 min
t2	30 min of post-saline infusion recording	60 min
t3	End of acid infusion	90 min
t4	15 min of post-acid infusion recording	105 min
t5	30 min of post-acid infusion recording	120 min
t6	Before the first saliva bolus	135 min
t7	Before the second saliva bolus	180 min
t8	End of recording	210 min

MII-pH: Multichannel intraluminal impedance and pH

To evaluate the influence of the two different infusions (saline and acid solution), percentage of decreases was calculated using the end of the baseline recording (t0) as 100%, according to the following formulas:

NaCl infusion= x : 100 = (t1-t0) : t0

HCl infusion= x : 100 = (t3-t0) : t0

On the other hand, to evaluate the MII-pH recovery 30 min after the end of each infusion, percentages were calculated using the difference between the recordings at the end of the infusions and the baseline as 100%, according to the following formulas:

NaCl infusion= x : 100 = (t2-t1) : (t0-t1)

HCl infusion= x : 100 = (t5-t3) : (t0-t3)

### Sampling for EB permeability assay and histology

At the end of the experimental protocol, each esophagus, including the three control ones, was longitudinally cut before ISFET probe removal and the exposed mucosa was carefully washed for 5 min in saline solution (NaCl 0.9%) for macroscopic observations. A full-thickness biopsy was performed 7 cm above the last impedance recording channel and divided into two pieces: One for EB permeability assay and one for histology.

### EB permeability assay

For the EB permeability assay, 500 µL of freshly prepared EB solution (10 mg/mL in NaCl 0.9% saline solution; Sigma-Aldrich, NJ, USA) were placed on top of each mucosa fragment, previously placed upward in a Petri dish. After 10 min, samples were washed twice in saline solution (NaCl 0.9%): The first wash consisted of two rapid dips in 100 mL of saline solution, while the second one, in 50 mL of saline, lasted 5 min. At the end, all samples were trimmed and divided into two aliquots to get technical duplicates. Aliquots were then dried at 37°C in a thermostatic hood for 30 min and the mucosal layer was isolated, immediately weighed, placed in 3 mL of formamide (Sigma-Aldrich, NJ, USA) and incubated for 48 h at 50°C to extract EB dye. Colorimetric measurements were performed using a spectrophotometer (Gene Quant 1300; GE Healthcare, UK) at the maximum absorption for EB (620 nm). Micrograms of EB per mg of tissue were quantified using a standard curve (0.025-25 μg/mL EB in formamide) and samples’ weights. Data of the two technical replicates were averaged.

### Histology

Samples for histology were fixed overnight in cold 4% formalin solution (in 0.1 M phosphate buffer, pH 7.4, Kaltek, Italy) and then moved into 25% sucrose (Sigma-Aldrich, NJ, USA) solution in phosphate buffer at 4°C for at least 24 h. Afterward, samples were embedded in OCT (Sakura Finetek, USA). Ten micrometer sections were cut at a Leica CM1950 cryostat (Leica, Wetzlar, Germany), mounted on microscope’s slides, and stained with hematoxylin and eosin (H&E) according to standard procedure. Images were obtained using a camera (Digital C-Mount Camera, Nikon, Tokyo, Japan) installed on an inverted microscope (ECLIPSE TS100, Nikon, Tokyo, Japan) using the Alexasoft X-Elit image analysis system (Alexasoft, Florence, Italy). For each slide, six measures were done to evaluate the thickness of the mucosal layer.

### Statistical analysis

Statistical analysis was performed using the software GraphPad Prism (GraphPad, La Jolla, CA, USA). The distribution of all data was assessed by means of Shapiro–Wilk test. Regarding MII-pH, to compare the percentages of decrease and increase between saline and acid infusions in the experimental esophagi (n=10), either paired t-test or Wilcoxon test was used. To evaluate differences between the baseline values (t0) and the different time points during the post-acid infusion recordings (from t3 to t8), a repeated measure (RM) ANOVA, followed by Tukey’s *post hoc* test, was performed. The results of EB permeability assay for control esophagi and acid-damaged ones were compared by means of Welch’s *t*-test, while for histological data, Mann–Whitney’s *U*-test was applied. Significance was set for p<0.05 (Confidence Interval 95%).

## Results

All the used esophagi did not show any macroscopically visible alteration, on evaluation of the mucosa after longitudinal cutting, imputable to both pre-existing condition and the experimental protocol itself. Raw data of the different esophagi were averaged. At the end of each infusion, the entire volume of the used solution was recovered, yet with small temporal differences: Saline and acid solutions were completely recovered within 15 s from the end of infusion, while saliva within 40 s.

### MII-pH

**Figure-2:**
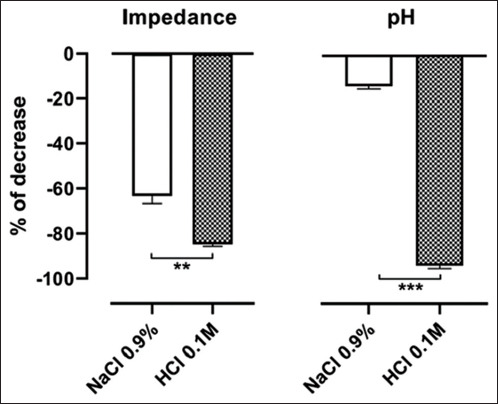
Percentages of decrease in MII and pH after saline (NaCl 0.9%) and acid (HCl 0.1 M) infusions in comparison to the baseline values. Data are reported as means and standard error of the mean. (Impedance: Paired *t*-test; pH: Wilcoxon test; **p<0.01; ***p<0.001).

For each esophagus, the impedance recordings of the six channels of the ISFET probe were averaged. The effects of the infusion of saline (NaCl 0.9%; t1) and acid (HCl 0.1 M; t3) solutions on MII-pH are represented in Figure-2 as percentage of decrease in comparison to the baseline values (t0). The decrease in both parameters induced by the acid solution was statistically more marked when compared to the one induced by saline solution.

**Figure-3:**
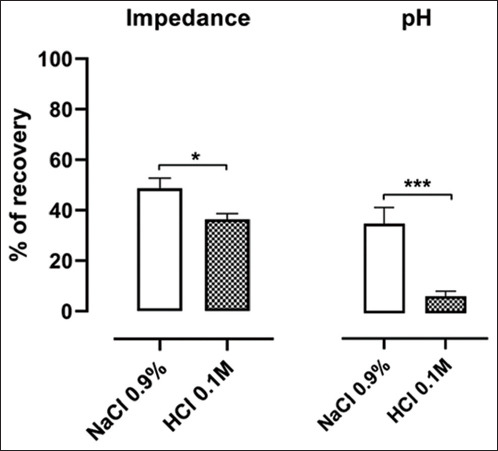
Percentages of recovery in multichannel intraluminal impedance and pH 30 min after saline (NaCl 0.9%) and acid (HCl 0.1 M) infusions. Data are reported as means and standard error of the mean (paired *t*-test; *p<0.05; ***p<0.001).

The percentages of MII-pH recovery 30 min after the end of each infusion (NaCl 0.9% t2; HCl 0.1 M t5) are represented in Figure-3. Also, in this case, both parameters show a statistically significant difference when comparing the two solutions, with better recovery recorded after the saline infusion.

**Figure-4:**
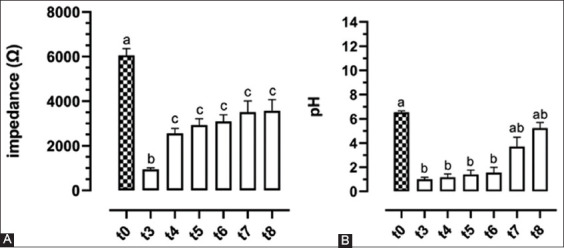
Multichannel intraluminal impedance (A) and pH (B) values at different time points after acid solution (HCl 0.1 M) infusion in comparison to the baseline (t0). Different letters indicate statistically significant differences between time points (RM ANOVA, Tukey’s *post hoc* test; p<0.05). t3=end of acid infusion; t4=15 min of post-acid infusion recording; t5=30 min of post-acid infusion recording; t6=before the first saliva bolus; t7=before the second saliva bolus; t8=end of recording.

The results of the RM ANOVA for the evaluation of absolute MII-pH values at different time points after the acid solution (HCl 0.1 M) infusions are represented in Figure-4. When compared to the baseline (t0), MII-pH values at the end of the infusion (t3) were statistically lower. After 15 min of post-infusion recording (t4), MII significantly increases and remains constant without ever reaching the baseline values. On the other hand, pH values remain low and only increase after the second saliva bolus (t7), reaching statistically similar values when compared to the baseline (t0).

### EB permeability assay

**Figure-5:**
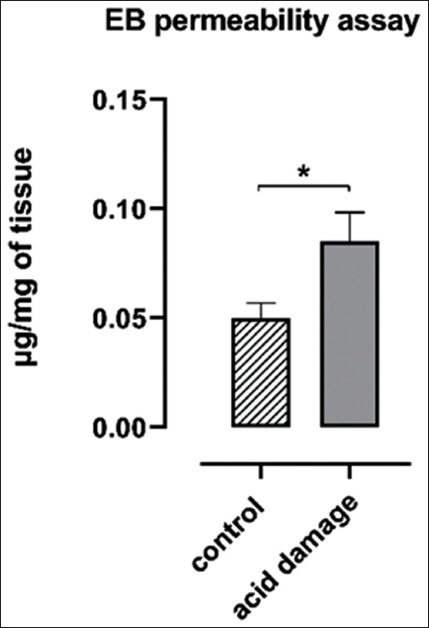
Evans blue permeability assay on control (n=3) and experimental (n=10) esophagi. Data are reported as means and standard error of the mean (Welch’s *t*-test, *p<0.05).

The results of the comparison of the EB permeability assay performed on control (n=3) and experimental (n=10) esophagi are represented in Figure-5. A statistically significant increase was recorded for the samples obtained by the esophagi that underwent the experimental protocol and were, therefore, exposed to the acid solution (HCl 0.1 M).

### Histology

**Figure-6:**
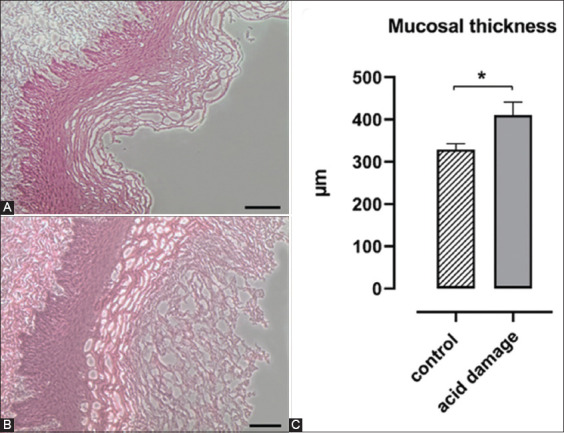
Hematoxylin-eosin staining of control (A) and experimental (B) esophagi (100×; bars: 100 μm). Comparison of mucosal thickness between the two groups (C). Data are reported as mean and standard error of the mean (Mann–Whitney *U*-test, *p<0.05).

The results of the histological analysis of the mucosal thickness on control (A; n=3) and experimental (B; n=10) esophagi and their comparison are represented in Figure-6. As for the EB, a statistically significant increase in mucosal thickness was recorded for the samples obtained by the esophagi that underwent the experimental protocol and were, therefore, exposed to the acid solution (HCl 0.1 M).

## Discussion

As previously stated, the aim of the work was to create an *ex vivo* model capable of mimicking esophageal acid reflux for preliminary screening of medical devices and therapies. It is indeed extremely important to scrutinize and develop preclinical models to reduce the number of animals enrolled in *in vivo* studies. In such scenario, the possibility to use organs collected at slaughterhouses represents a good option in full respect of the 3Rs principle [23,24] and has already been described for the porcine species [11,25]. Nonetheless, such collection procedure has some limitations including the lack of sterility and the necessity for trained personnel capable of noticing potential lesions caused by slaughter itself.

All of the esophagi used for the experimental protocol showed basal levels of MII-pH similar to the ones recorded in healthy adult humans in physiological conditions [26]. This finding is important as it validates the already known anatomical/functional similarities of pigs when compared to humans. Indeed, in the past decades, this species has emerged in the field of translational medicine, especially when it comes to gastroenterology. This is potentially related to the fact that, out of the most commonly used large animal models, pigs are non-ruminant omnivorous and show metabolic patterns close to humans.

The same model was already proposed and used to evaluate anti-GERD devices by the same research group [11], but this work shows the addition of a better definition of the damage by means of a quantitative evaluation of EB permeability assay and of a well-recognized method in human gastroenterology as MII-pH analysis. In physiological conditions, when the mucosal layers are intact, electrical impedance is high and only drops when liquids wet the above-mentioned layers. On the other hand, in case of anatomical alterations of the mucosa such as erosive lesions, the baseline impedance is lower [27,28]. When it comes to GERD, MII-pH is used to identify and classify reflux episodes: A reflux episode is defined as a fall in impedance of ≤50% of baseline and, when pH lowers and remains ≤4 for at least 5 s, can be considered acid [29]. According to the results, it can be stated that the infusion of acid solution, HCl 0.1 M in this peculiar case, has indeed determined the simulation of an acid reflux event.

One of the downsides of the proposed experimental model is the lack of the physiological peristaltic movements characteristic of the esophageal functionality. Unfortunately, such statement can be applied to the majority of the currently available *ex vivo* models, and work needs to be done to implement this gap potentially by means of mechanical devices. Nonetheless, the capability to recover the entire volume of the infused solutions from the lower end of the organs indicates a correct flow within the esophagi and the absence of liquids accumulation, also confirmed by MII-pH. The temporal delay observed for the recovery of the saliva when compared to the other solutions has to be imputed to the viscosity of the artificial saliva and further supports the sensitivity of the model.

The impedance results show how it dropped, as expected, during every infusion, proving not only the sensitivity of the recording itself but also most important that the probe was always in contact with the mucosal layer. The latter is pivotal for a correct recording and having the chance to visualize the recording “live” is pivotal in such trials. Nonetheless, not all the commercially available impedance recorders offer this feature, mainly to preserve the battery’s autonomy during 12-24 h recording. For this particular trial, a modified impedance recorder was developed to allow a battery autonomy of at least 4 h with active screen. The sensitivity of the method is further confirmed by the fact that the decrease in MII determined by the acid solution was statistically more severe, indicating that what is registered cannot only be imputed by the wetting of the mucosal layer but also by the nature of the used solution.

After the two impedance drops determined by saline and acid solution infusion, the patterns of recovery were statistically different, with a faster and more marked recovery after the infusion of saline. This result seems to confirm the hypothesis of acid-induced damage to the mucosa, that is, not capable of completely restoring the normal baseline electric impedance. To further support such statement, the results of the RM ANOVA analysis (Figure-4A) show how MII increases within the first 15 min after the acid infusion, but immediately reaches a plateau thereafter (from t4 to the end of the recording) and never gets back to normal values despite the two saliva boluses.

All the discussions regarding MII are strengthened by the results of pH (Figure-4B), recorded by the same probe (MII-pH) and the same recorder. Being the pH of the used solution standardized, the results are coherent with the anticipated outcome. Indeed, the saline solution (NaCl 0.9%) always induced statistically milder pH variations when compared to the ones induced by the acid solution. Nonetheless, as opposite to MII, the pH showed a better recovery during the 2 h of post-acid infusion recording, reaching levels comparable to the baseline at the last 2 time points. Such different behavior is imputable by the nature of the parameter itself, directly correlated to the used solutions rather than the integrity of the mucosa. In this case, the saliva boluses may have better contributed to the recovery as opposed to what happened for MII.

The results of EB permeability assay and histology confirm what recorded by MII and demonstrate the hypothesized acid-induced damage to the mucosa. EB is known to bind quantitatively to albumin *in vivo* and *in vitro* and so it could be used as an indicator of esophageal mucosa permeability [30-32]. A previous experiment with the same model showed that the absorption of EB was due to the increased mucosal permeability caused by the acid perfusion since the intact mucosa did not absorb the dye [11]. To obtain a more objective measurement of the absorption, unlike the previous experiment, we used a method of extraction and quantification of the dye instead of the visual analysis of the staining. The comparison between the 10 experimental esophagi and the 3 control ones highlighted how the acid solution infusion allowed higher amounts of EB to permeate into the sample. In physiological conditions, an intact mucosal layer should almost completely prevent stains like EB to penetrate the deeper tissue layers, as seen in the control organs. The higher concentrations of EB found in acid-treated esophagi are, therefore, indicative of increased mucosal permeability, again already suggested by the other results previously discussed. Such hypothesis was definitively confirmed by histology that showed an increase in mucosal thickness, due to exfoliation and loss of continuity.

## Conclusion

According to the results of the present study, the hereby proposed model of esophageal acid-induced damage seems to be repeatable, reliable, and achievable using organs collected at the slaughterhouse in respect of the 3Rs principles. MII recording, largely used in clinical settings, proved to have good sensitivity in detecting mucosal alterations also in *ex vivo* trials. Overall, the model may be suitable for preliminary screening of both drugs and medicals devices developed to treat GERD, in light of its relatively low ethical and economic impacts.

## Authors’ Contributions

MLB, MF, and FB conceived and designed the study. DV, AE, RS, and GC performed the experiments, and AE analyzed the data. DV, RS, and GC drafted the manuscript. All authors revised the final manuscript and approved it.
